# Functional Photocatalysts: Material Design, Synthesis and Applications

**DOI:** 10.3390/molecules29051146

**Published:** 2024-03-05

**Authors:** Lin Ju

**Affiliations:** School of Physics and Electric Engineering, Anyang Normal University, Anyang 455000, China; julin@aynu.edu.cn

Rapid industrial and economic growth, experienced on a global scale, has been greatly facilitated by the extensive use and exploitation of traditional energy resources. However, this progress has also resulted in significant environmental pollution and energy scarcity issues, which pose serious threats to our living environment and the future advancement of humanity. Consequently, many researchers are now shifting their focus towards exploring and developing new clean energy solutions, with the aim of addressing the escalating concerns of environmental degradation and energy deficits [1,2,3,4]. Among these solutions, solar energy stands out as an endless and renewable power source. Its further application in tackling the current environmental crises could represent a remarkable step forward for the collective well-being of humanity worldwide. Therefore, the progress of solar energy technology carries profound implications [5,6,7]. Semiconductor photocatalysis is a defined process that utilizes solar energy to initiate excitation in semiconductors, generating photogenerated carriers. These carriers subsequently promote catalytic redox reactions on the surface of the semiconductor. This innovative approach not only enables the transformation and storage of solar energy but also opens possibilities for conversion into various forms of energy [8,9,10,11].

In this Special Issue, our focus is centered on the design, synthesis, and diverse applications of functional photocatalysts. We have curated a collection of ten high-quality papers that are poised to capture the interest of researchers in the field of photocatalysis. Among them, four papers present a comprehensive examination of CO_2_ conversion methods, two explore the synthesis of NH_3_, one is dedicated to investigating the water splitting process, another delves into the degradation of organic pollutants, while the final two papers focus on the development of promising heterojunction photocatalysts. We hope that this Special Issue will act as a catalyst for further advancements in photocatalytic technologies, spurring progress in the generation of new energy sources and the remediation of environmental pollution.

**CO_2_ reduction reaction (CO_2_RR)**. 

In recent years, the depletion of fossil fuel reserves and the rise in atmospheric CO_2_ levels have underscored the urgency of developing sustainable solutions for converting excess CO_2_ into valuable chemicals and fuels [12,13,14]. This not only addresses issues such as the greenhouse effect, the melting of glaciers, and other environmental challenges associated with carbon dioxide, but also provides a potential remedy for the ongoing energy crisis [15]. Carbon dioxide conversion can be achieved through diverse methods, including biochemical [16], electrochemical [17,18], photochemical [19,20], and thermochemical [21] processes. Solar-powered CO_2_ reduction, harnessing sunlight as an inexhaustible energy source, has emerged as the most promising approach among these methods [22,23]. Therefore, it has attracted considerable attention, yielding significant advancements [24,25,26,27]. Through first-principles calculations, Ju et al. (contribution 1) discovered that introducing selenium vacancies leads to a transition from physical to chemical CO_2_ adsorption on Janus WSSe nanotubes. These Se vacancies serve as effective adsorption sites, significantly boosting electron transfer at the interface. This results in heightened electron orbital hybridization between adsorbents and substrates, thereby promising elevated activity and selectivity in CO_2_RR. Under illumination, photoexcited holes and electrons generate adequate driving forces, enabling simultaneous oxygen generation reaction (OER) and producing CO_2_RR on the sulfur and selenium sides of the defective WSSe nanotube, respectively. This process allows for the reduction of CO_2_ into methane, while O_2_ is produced through water oxidation, which also supplies hydrogen and electrons for CO_2_RR. The findings unveil a potential photocatalyst for achieving efficient photocatalytic CO_2_ conversion. Wang et al. (contribution 2) developed an innovative dinuclear Gd (III) complex. This new compound is distinguished by its intricate three-dimensional π–π stacking network, where the voids are occupied by chloride anions and water molecules that are not coordinated. A detailed examination of the complex’s Hirschfeld surface indicates the major presence of H···H interactions, constituting 48.5% of the surface interactions. These are followed by C···H/H···C and O···H/H···O interactions, contributing 27.2% and 6.0%, respectively. The complex showcases notable efficiency in photocatalytic CO_2_ reduction experiments, yielding 22.1 µmol/g of CO and 6.0 µmol/g of CH_4_ within a span of three hours, with an impressive 78.5% selectivity for CO production. This research sheds light on new possibilities for advancing the study and synthesis of rare earth metal complexes, particularly in the realm of photocatalytic activities for CO_2_ reduction. Yuan et al. (contribution 3) delved into the latest progress in solar-powered CO_2_ hydrogenation, emphasizing catalyst designs, the architecture of active sites, and the underlying mechanisms. The quest to enhance catalytic efficiency and tackle the hurdles associated with CO_2_ reduction has led to the emergence of various innovative strategies. Key among these is the harnessing of light energy, achieved by amplifying the light absorption capacity of catalyst materials. This is primarily facilitated by localized surface plasmon resonances triggered by metal particles (such as Pd, Rh, Ni, Co) and the creation of vacancies. The inclusion of metal particles not only augments optical absorption but also furnishes vital active sites for the activation of H_2_ and CO_2_ molecules. Furthermore, the introduction of anion vacancies plays a crucial role in surface catalytic reactions by bolstering CO_2_ adsorption and diminishing the activation energy required for its reduction. Advanced nanostructures, including photonic crystals, have been meticulously engineered to optimize light utilization. Notably, indium-based oxides, distinguished by their versatile morphologies, phases, and surface-active sites, have demonstrated significant potential in CO_2_ hydrogenation. Despite these advancements, several challenges persist, including the limited catalytic activity of numerous catalysts, their prohibitive costs, the notable scarcity of their industrial-scale production, and the difficulties developing specialized apparatus. Moreover, among various photocatalysts, transition metal oxide Cu_2_O stands out for its narrow band gap, effective visible light absorption, appropriate conduction band level, affordability, and significant photocatalytic potential. Su et al. (contribution 4) delve into Cu_2_O’s fundamental characteristics, fabrication techniques, and enhancement approaches. They survey recent Cu_2_O-based photocatalysts, as well as their advancements in CO_2_ reduction and other applications. The review also points out areas for improvement in Cu_2_O-based materials, such as the reliance on costly noble metals for composite and sacrificial agent synthesis, hindering widespread use; challenges in mass-producing high-quality photocatalysts due to potential nanomaterial risks; ongoing issues with photocorrosion that impact durability; and the yet-to-be-clarified catalytic structures and mechanisms of Cu_2_O composites.

**NH_3_ synthesis**. 

Ammonia, vital in both modern chemical applications and as a structural component in biological molecules, is largely produced industrially via the Haber–Bosch (H-B) method [28]. This method synthesizes ammonia by catalyzing a reaction between nitrogen and hydrogen at elevated temperatures and pressures using metal catalysts [29,30]. Although effective, the H-B method consumes considerable energy and contributes substantially to carbon emissions. To counter this, the “double carbon” initiative promotes combining photocatalytic technologies with synthetic nitrogen fixation, aiming for more sustainable synthetic processes. Researchers harness the sun’s abundant energy, using photocatalysts to transform nitrogen/oxynitride into ammonia, a process noteworthy for its energy-saving attributes and simplified storage and transport [31,32]. This approach signifies a shift from solar to chemical energy, paving the way for zero carbon emissions, and thus drastically curbing energy use and environmental impact in industrial ammonia production. Photocatalytic methods in the nitrogen cycle are gaining traction as a significant research field in renewable energy. Zuo et al. (contribution 5) primarily discuss the utilization of composite materials in the field of photocatalytic nitrogen fixation. Their research emphasizes the process of creating NH_3_ from N_2_ and H_2_O using solar power as a renewable energy source under gentle conditions. The study investigates several approaches, including the introduction of defects, the formation of heterojunctions, and the doping of elements, that can be used to adjust the semiconductors’ band gap width. These methods aim to enhance the semiconductor’s sensitivity to visible light and optimize the usage of light energy. Furthermore, the review introduces the importance of enhancing the movement and separation of photogenerated electrons and holes within the catalysts. This improvement is crucial for increasing the lifespan of these photogenerated carriers and boosting the quantum efficiency of photocatalytic processes. Besides, the photocatalytic NO reduction reaction (NORR) is considered to be a dual-purpose method for both NO removal and NH_3_ production. This highlights the necessity for the significant activation of NO molecules by the catalyst, which requires effective chemisorption. Using first-principles calculations, Ju et al. (contribution 6) report a notable transition from physical to chemical adsorption of NO molecules on the Janus WSSe monolayer after introducing Se vacancies. These vacancies potentially serve as optimal adsorption sites, substantially enhancing the electron transfer at the interface. This leads to a pronounced hybridization of electronic orbitals between the adsorbate and the substrate, indicating high NORR activity and selectivity. Furthermore, the spatial constraints imposed by the Se vacancy defects effectively inhibit the N≡N bond coupling and *N diffusion in NO molecules, thus endowing the active site with superior selectivity for NORR in NH_3_ synthesis. Additionally, their study reveals that the photocatalytic conversion of NO into NH_3_ can spontaneously occur, driven solely by the photo-generated electrons. These insights pave the way for developing highly efficient photocatalysts for NO-to-NH_3_ conversion.

**Water splitting**. 

Semiconductor-based photocatalytic water splitting for hydrogen production has gained prominence in sustainable energy research [33,34,35,36], especially since the initial discoveries involving TiO_2_ photocatalysts [37]. The advent of two-dimensional materials has furthered this field, offering novel photovoltaic and photocatalytic solutions due to their superior properties like extensive specific surface areas, numerous active sites, and reduced carrier migration distances. Huang et al. (contribution 7) examine four δ-IV–VI monolayers, namely, GeS, GeSe, SiS, and SiSe, by employing the first-principles method. These monolayers are noted for their remarkable resilience, with the GeSe monolayer displaying consistent yield strength, even at 30% strain. Notably, the GeSe monolayer boasts an extraordinarily high electron mobility along the x-axis, around 32,507 cm^2^·V^−1^·s^−1^, significantly surpassing that of its δ-IV–VI counterparts. Additionally, these monolayers’ potential in hydrogen evolution reaction capacity, as calculated, suggests promising applications in the realm of photovoltaic and nano-devices.

**Degradation of organic dye**. 

The growth of the textile industry presents notable environmental challenges, especially due to the release of organic dye waste. This wastewater normally contains dyes, such as methyl orange (MO), crystal violet (CV), Congo red (CR), methylene blue (MB), and metanil yellow (MY), or combinations thereof. These dyes, infiltrating water sources for drinking, pose carcinogenic threats to human health [38]. Employing nanomaterials like semiconductors for the photocatalytic breakdown of such dye contaminants offers a potential solution [39]. Wang et al. (contribution 8) utilize a mechanochemical activation-assisted solid-state reaction (MAS) to create LuFe_1−x_Co_x_O_3_ powders (where x = 0, 0.05, 0.1, 0.15). Investigations using X-ray diffraction (XRD) and Fourier transform infrared (FTIR) spectroscopy reveal that the substitution of B-site iron ions with cobalt ions leads to reductions in lattice parameters. The powder’s morphology and elemental composition are further analyzed using scanning electron microscopy (SEM) and energy-dispersive spectroscopy (EDS). UV–visible absorption spectra indicated that LuFe_0.85_Co_0.15_O_3_ powders exhibit a narrower bandgap of 1.75 eV and greater absorbance compared to LuFeO_3_ (2.06 eV), significantly enhancing light absorption efficiency. Moreover, the LuFe_0.85_Co_0.15_O_3_ powders displayed superior photocatalytic ability over LuFeO_3_, demonstrating near-complete degradation of methyl orange (MO) in 5.5 h under visible light irradiation with oxalic acid aid. These findings underscore that cobalt doping can notably enhance the photocatalytic efficiency of orthorhombic LuFeO_3_ for organic dye degradation.

**Heterostructures for photocatalysts**. 

The use of semiconductor-based photocatalysis is garnering significant interest for its potential to harness solar energy directly in order to produce solar-based fuels such as hydrogen and hydrocarbons, in addition to its role in breaking down various pollutants. Despite its promise, the current effectiveness of photocatalytic processes is limited due to rapid recombination of electron–hole pairs and the suboptimal utilization of light. Addressing these challenges has become a focus of substantial research efforts. In particular, the development of specially designed heterojunction photocatalysts has demonstrated enhanced photocatalytic performance, attributable to the effective spatial separation of photogenerated electron–hole pairs [40,41]. Obtaining precision in cultivating or assembling intricate heterostructures remains a considerable challenge. Zhang et al. (contribution 9) explore this issue by examining the collision dynamics of carbon and boron nitride nanotubes under varied collision scenarios, employing self-consistent-charge density-functional tight-binding molecular dynamics. Their study, involving first-principles calculations, reveals the energy stability and electronic structures of heterostructures post-collision. The authors identify five primary collision outcomes: nanotubes can either (1) bounce off each other, (2) join together, (3) merge into a larger, defect-free boron–carbon–nitrogen (BCN) heteronanotube, (4) form a heteronanoribbon combining graphene and hexagonal boron nitride, or (5) suffer severe damage. Notably, both the defect-free (12, 0) BCN heteronanotube and the BCN heteronanoribbon are found to be direct band-gap semiconductors, with band gaps of 0.808 eV and 1.34 eV, respectively. These findings suggest that the electronic structures of nanotubes can be effectively altered through the collision of carbon and boron nitride nanotubes, potentially impacting photocatalytic and other photo-electric applications. While the study initially focuses on the collision fusion of CNTs and BNNTs with specific helicity, its methodology could be applicable to nanotubes of any helicity. Their work not only theoretically introduces a novel method for creating heterostructures via collision fusion but also provides a deeper understanding of the synthesis of heteronanotubes and heteronanoribbons, offering guidance for experimental endeavors. Furthermore, Bissenova et al. (contribution 10) develop a hybrid structure comprising anodic TiO_2_ nanotubes intermixed with SrTiO_3_ particles through chemical synthesis methods. The TiO_2_ nanotubes are fabricated by anodization in a solution of ethylene glycol containing NH_4_F and H_2_O, with a voltage of 30 volts applied. Subsequently, a nanotube array annealed at 450 °C is submerged in a dilute SrTiO_3_ solution within an autoclave. Scanning electron microscopy (SEM) analysis reveals that the titanium nanotubes are characterized by clear and open ends, boasting an average exterior diameter of 1.01 µm, an interior diameter of 69 nm, and a length measuring 133 nm. Their findings verify the successful creation of a composite structure with potential utility in numerous fields, notably for hydrogen generation through the photocatalytic splitting of water under solar illumination.

It is our sincere hope that the articles published in this Special Issue will contribute to innovation and further in-depth research in the field of photocatalysis. The insights and perspectives presented in each article demonstrate the research results achieved under distinct scenarios. With the impetus of this Special Issue, we eagerly look forward to the emergence of breakthrough research in this field, which will play a key role in addressing the energy crisis and solving the problem of environmental pollution.
